# Rapid Evolution of Virus Sequences in Intrinsically Disordered Protein Regions

**DOI:** 10.1371/journal.ppat.1004529

**Published:** 2014-12-11

**Authors:** Leonid Gitlin, Tzachi Hagai, Anthony LaBarbera, Mark Solovey, Raul Andino

**Affiliations:** Department of Microbiology and Immunology, University of California, San Francisco, San Francisco, California, United States of America; Virginia-Maryland Regional College of Veterinary Medicine, United States of America

## Abstract

Nodamura Virus (NoV) is a nodavirus originally isolated from insects that can replicate in a wide variety of hosts, including mammals. Because of their simplicity and ability to replicate in many diverse hosts, NoV, and the *Nodaviridae* in general, provide a unique window into the evolution of viruses and host-virus interactions. Here we show that the C-terminus of the viral polymerase exhibits extreme structural and evolutionary flexibility. Indeed, fewer than 10 positively charged residues from the 110 amino acid-long C-terminal region of protein A are required to support RNA1 replication. Strikingly, this region can be replaced by completely unrelated protein sequences, yet still produce a functional replicase. Structure predictions, as well as evolutionary and mutational analyses, indicate that the C-terminal region is structurally disordered and evolves faster than the rest of the viral proteome. Thus, the function of an intrinsically unstructured protein region can be independent of most of its primary sequence, conferring both functional robustness and sequence plasticity on the protein. Our results provide an experimental explanation for rapid evolution of unstructured regions, which enables an effective exploration of the sequence space, and likely function space, available to the virus.

## Author Summary

Proteins often contain regions with defined structures that enable their function. While important for maintaining the overall architecture of the protein, structural conservation adds constraints on the ability of the protein to mutate, and thus evolve. Viruses of eukaryotes, however, often encode for proteins with unstructured regions. As these regions are less constrained, they are more likely to accumulate mutations, which in turn can facilitate the appearance of novel functions during the evolution of the virus. Even though it has been known that such “disordered protein regions” have been particularly malleable in evolution, their functions and their ability to withstand extensive mutations have not been explored in detail. Here, we discovered that a disordered part of the Nodamura Virus polymerase is both required for replication of the viral genome, and extremely variable among different nodaviruses. We examined the tolerance of this protein region to mutations and found an unexpected ability to accommodate very diverse protein sequences. We propose that disordered protein regions can be a reservoir for evolutionary innovation that can play important roles in virus adaptation to new environments.

## Introduction

Nodamura virus (NoV) is the founding member of the family *Nodaviridae*. Viruses of this family combine several remarkable features. First, they are capable of replicating in a great variety of diverse hosts. While their natural hosts are insects (in the case of alphanodaviruses) and fish (for betanodaviruses), RNA from NoV and Flock House virus (FHV) is capable of replication in plant, yeast, and mammalian cells [1]–[4]. Furthermore, their genomes are among the smallest known (NoV genome is 4.5 kb long), and are split between two segments, called RNA1 and RNA2. RNA1's ORF A encodes protein A, which contains the viral RNA-dependent RNA polymerase (RDRP), but is likely to possess other activities as well, such as capping of viral RNAs [5]. RNA2 encodes the capsid protein alpha. RNA2 is not required for viral RNA replication. Indeed, RNA1 can replicate autonomously when introduced into cells [2]. Thus, at 3.2 kilobases in length, RNA1 represents one of the smallest animal virus replicons which encodes its own polymerase.

**Figure 1:**
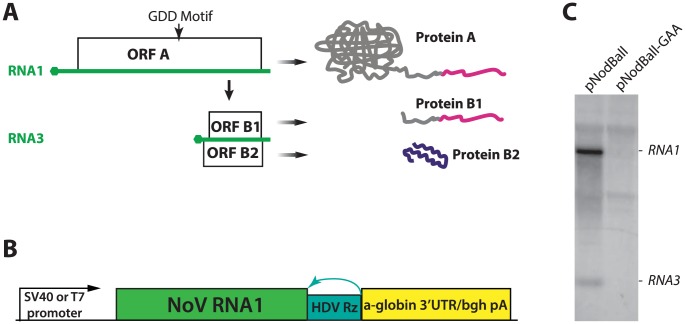
A. A schematic depiction of NoV RNA1 and its translation and replication products. RNA is shown in green; hexagons indicate the cap structure. ORF A is translated from RNA1 and initiates replication of RNA1 and RNA3. ORF B1 is equivalent to the C-terminus of ORF A, while ORF B2 is frameshifted with respect to A and B1. The extreme C-terminal fragment of proteins A and B1 (A^C-TERM^), which is mutated below (See Fig. 7), is shown in magenta. Also, the location of the GDD motif of the polymerase is shown; the GDD→GAA mutant is used as a non-replicating control throughout this work. **B.** Design of the constructs used to express NoV RNA1 and replicons. See text for detailed description. **C.** Northern analysis of plasmid transfection. Left lane: plasmid encoding wildtype (GDD) polymerase; right lane: plasmid encoding a control (GAA) polymerase. The background bands common to both lanes correspond to rRNAs.

During replication, RNA1 gives rise to a subgenomic RNA, called RNA3 (Fig. 1A). This 473-nucleotide sequence is identical to the 3′ end of RNA1, and can translate two different products from two overlapping ORFs. B1 is a protein produced from the same frame as protein A, and therefore represents the C-terminus of protein A. B2, on the other hand, is encoded by an overlapping, +1 frameshifted ORF [6]. Remarkably, the reading frames run alongside each other for the entire length of B2 (see Fig. 1A). B2 is required for nodavirus replication for at least two reasons: first, it enables RNA2 translation [7], and second, it blocks the antiviral RNAi response in insect cells [8], [9]. RNA1 replication in interferon-deficient mammalian cells, however, does not require B2; thus, its ORF can be removed from RNA1-based replicons with minimal consequences for replication [10].

In summary, three features of NoV combine to set it apart: (1) The compactness of its genome, (2) the self-contained replication apparatus with minimal demands on the host, and (3) the ability to replicate its RNA in the absence of several viral proteins. These features make NoV an ideal platform for understanding critical requirements for replication of eukaryotic viral RNA. In addition, NoV represents a simple and attractive model for studying virus biology, for assessing host responses to a viral pathogen, and for engineering simple expression vectors. We therefore set out to develop NoV RNA1-based replicons which can express foreign genes.

In the process of engineering NoV replicons, we identified an RNA element mapping around the stop codon of ORF A, which is required for efficient RNA replication. We also found that the nodavirus-specific C-terminus of NoV proteins A and B1, which we call A^C-TERM^ (depicted in magenta in Fig. 1A), and which has not been characterized, is essential for replication of viral RNA. Strikingly, A^C-TERM^ can be replaced by completely unrelated amino acid sequences as long as they contain a certain arrangement and a minimum number of positive charges. This terminal region of the polymerase is predicted to be disordered ([5], also see below). Disordered regions in many proteins are segments that do not fold stably into 3-dimensional domains but rather remain unstructured and are highly flexible, exerting their effects via short peptide motifs [11]–[13]. These regions are widespread, enriched in certain viral proteins [14]–[17], and play important roles in mediating regulatory protein-protein [18]–[21] and protein-nucleic acid interactions [22], [23]. While disordered regions are important in regulating cellular [24], [25] and viral functions [11]–[13], [26], the relaxed sequence and structural requirements placed on them are likely to make them amenable to rapid evolutionary adaptation. The relatively high evolutionary rate [27]–[29] associated with these regions can thus facilitate the rise of novel functions.

Here, we demonstrate that the C-terminus of nodaviral protein A is highly variable and provide experimental evidence that only a few positively charged residues within an unstructured region can preserve its essential function in virus replication. Strikingly, the sequence of this region evolves rapidly, incorporating changes that may be neutral or result in novel adaptive functions. Our observations support the concept that disordered regions within essential viral proteins expand the sequence and function space accessible to the virus. We propose that these regions can rapidly gain new functions (for example, form new protein-protein interactions) during viral adaptation to a changing environment.

## Results

### Construction of NoV replicons

We used NoV virus stocks to construct cDNA clones derived from RNA1 and RNA2. RNA1 was cloned into a plasmid (pNodBall) such that it was driven by the SV40 promoter and trailed by an HDV ribozyme (Fig. 1B). This approach, similar to the one previously used in FHV [30] allowed production of replication-competent RNA1 transcript without the need for T7 RNA polymerase-expressing cell lines used so far with NoV [10]. (For details on NoV cDNA derivation, please see Supporting Information).

In order to examine replication of RNA1 launched from plasmid DNA, we transfected BSR hamster kidney cells with pNodBall, isolated total RNA at 20 hours post-transfection, and analyzed it by Northern blotting (Fig. 1C). NodBall RNA replicon accumulated over time, while transfection with control NodBall-GAA (Fig. 1C, right lane) in which the “GDD” motif of the polymerase was replaced with the inactive amino acid sequence GAA [31], did not generate detectable replicon RNA. We conclude that wildtype RNA1 replicated efficiently and, as expected, also gave rise to RNA3 (Fig. 1C, left lane).

### Expression of reporter genes as cleavable fusions of NoV protein A

We next introduced several reporter genes into NodBall RNA1s. Protein B2 is required for viral RNA replication in insects as it suppresses antiviral RNAi [8]. It is, however, dispensable for viral RNA replication in yeast and interferon-deficient mammalian cells, though replication is slightly decreased [10]. To simplify the replicons we either deleted or truncated B2. We either fused the reporter gene to the C-terminus of protein A (“protein A fusions”) or to the C-terminus of a truncated protein B2 in the other frame (“protein B2 fusions”). Replacement of B2 portions with reporter genes which truncated protein A did not result in replication-competent replicons (not shown). In contrast, attaching GFP to the C-terminus of protein A was previously reported in Flock House virus [32]; we therefore chose this strategy to generate NoV replicons carrying foreign genes fused to the C-terminus of protein A. In our design, we additionally made use of the foot-and-mouth disease virus (FMDV) 2A peptide, which induces co-translational “self-cleavage” to release the foreign protein from the rest of protein A ([33], also see below).

**Figure 2:**
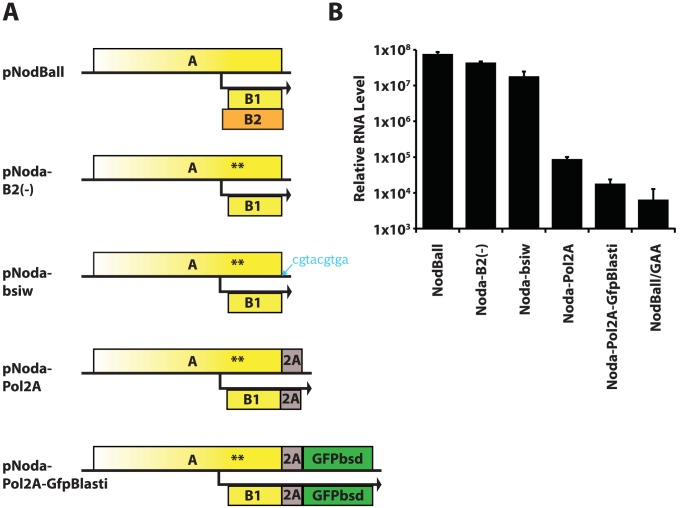
A. Initial replicon design strategy. The wt RNA1 is modified sequentially by truncation of the B2 ORF (nonsense codons in B2 are synonymous in A and B1 and are shown by asterisks); by insertion of 9 nt following the stop codon of ORF A; by cloning FMDV 2A sequence in frame with ORFA; and by cloning GFPbsd fusion gene in frame with A-2A. **B.** Replication efficiency of the replicons shown in Fig. 2A.

Initially, we created a series of constructs with modified 3′ end of RNA1. These constructs are schematized in Fig. 2A. First, we generated a B2-defective replicon pNodaB2(-) containing two premature stop codons in the B2 ORF shortly after the second AUG. This modification does not alter the amino acid sequence of protein A, but eliminates B2 production. In agreement with the literature [10], we find that B2 is not necessary for RNA1 replication in mammalian cells, as assessed by quantitative RT-PCR (Fig. 2B). We next inserted a BsiWI cloning site immediately downstream of ORF A, producing pNoda-bsiw. This minor alteration nevertheless lowered replication efficiency by 2–3 fold (Fig. 2B). When the 2A sequence derived from FMDV was fused to the C-terminus of protein A, we observed a further reduction in RNA1 (Fig. 2B, pNoda-Pol2A). The inserted 2A peptide is 24 amino acids long - it extends protein A by 23 C-terminal amino acids (and adds an N-terminal proline to any C-terminal transgene). RNA1 levels dropped even more when we introduced GFPbsd, a fusion of GFP and blasticidin resistance gene ORFs [34] (Fig. 2B, pNodaPol2A-GFPbsd), downstream of 2A.

### Optimization of NoV replicons

Because these progressive changes altered both RNA and protein sequence, we hypothesized that both RNA1 3′ end and protein A C-terminus are important for efficient RNA1 replication. To facilitate assessment and quantification of replicon efficiency, we generated new replicons, called pNoda-Pol2A-GFP and pNoda-Pol2A-Luc. These constructs are identical to pNoda-Pol2A-GFPbsd ones, with the exception of the GFPbsd reporter gene, which was replaced with GFP or the firefly luciferase gene, respectively.

**Figure 3:**
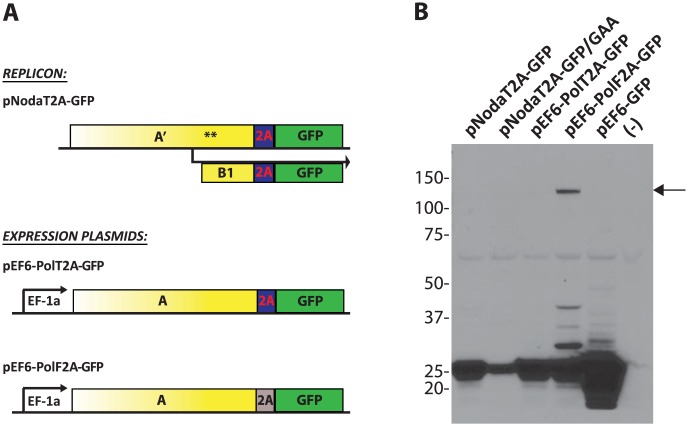
Processing of the GFP transgene by 2A cleavage peptides. **A.** Constructs used transfections. pNodaT2A-GFP replicon was either wt or contained the GAA polymerase-inactivating mutation. The other constructs are non-replicating Protein A-fusion expression plasmids. **B.** Co-translational “cleavage” efficiency of “T2A” and “F2A” peptides in the replicons and expression plasmids. The Western blot was probed for GFP. pEF6-GFP is used as a positive control; (-) denotes a lane with lysate from untransfected cells. Arrow indicates the unprocessed PolF2A-GFP polypeptide.

Next, we sought to improve polymerase activity of protein A fusion constructs. Since FMDV 2A (“F2A”) peptide-containing replicon was inefficient (Fig. 2B), we tested several other 2A sequences from other viruses [35]. We found that the *Thosea asigna* virus 2A (“T2A”) sequence allows for a modestly improved protein A expression (Fig. S1 in S1 Text). T2A sequence was shown to self-cleave very efficiently [36]. We verified that the transgene (GFP) is cleanly excised from the Pol2A-GFP replicons (Fig. 3). Pol2A-GFP expression plasmids demonstrated that T2A is indeed cleaved more efficiently than F2A (Fig. 3B). Consequently, T2A sequence was included in all subsequent constructs that employed protein A fusions.

### An RNA element inside ORF A augments RNA1 gene expression

**Figure 4:**
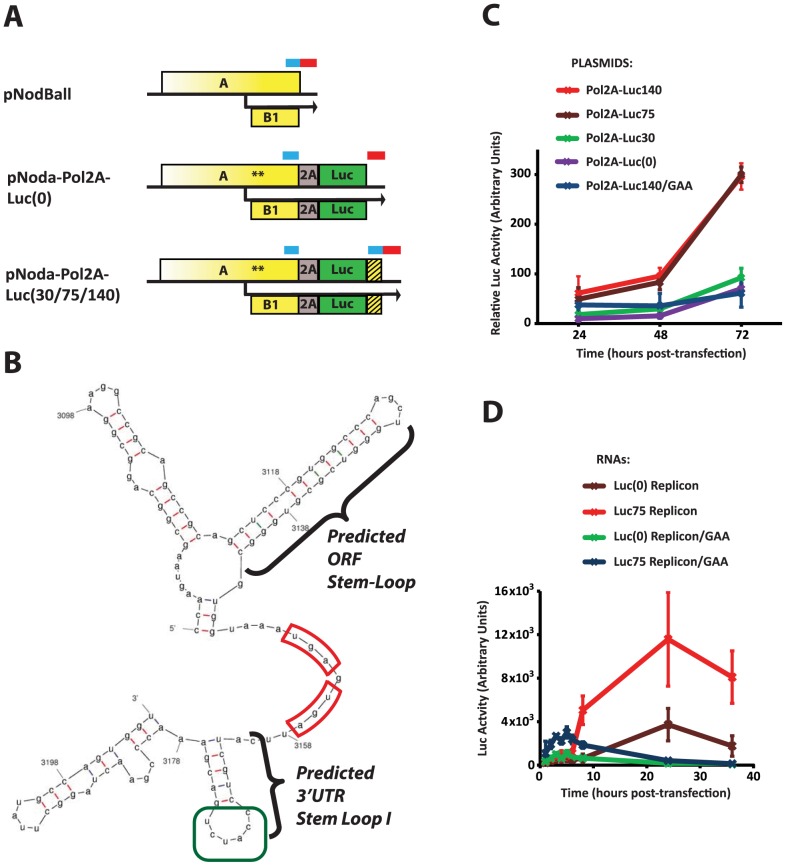
Identification of the ORF module of the 3′RE. **A.** Constructs used. pNodBall, similar to wt RNA1, contains intact 3′RE, consisting of the 3′UTR module (indicated with a red bar) and the ORF module (blue bar). Inserts such as pNoda-Pol2A-Luc(0) separate these modules, while the pNoda-Pol2A-Luc(30), pNoda-Pol2A-Luc(75) and pNoda-Pol2A-Luc(140) constructs reconstitute the various indicated lengths of the putative ORF module. The duplicated, untranslated ORF module is represented by the hatched box. **B.** Predicted structure of the 3′ end of NoV RNA1. MFold-generated structures consistently predict stem loops at the end of the ORFs and the 3′UTR stem loop I (highlighted). The stop codons for ORFs A and B2 are outlined in red; the CCAUCU sequence in the loop of the 3′UTR stem-loop I (see Discussion) is in green. **C.** Comparison of the luciferase counts from cells transfected with plasmids encoding the following replicons: Pol2A-Luc(0), Pol2A-Luc30, Pol2A-Luc75, and Pol2A-Luc140. Plasmid producing Pol2A-Luc140/GAA is used as a non-replicating RNA control. All firefly luciferase values were normalized by a co-transfected Renilla luciferase-expressing plasmid. **D.** Comparison of the luciferase counts from cells electroporated with the *in vitro* transcribed replicons. Replicons Pol2A-Luc(0) and Pol2A-Luc75 were used along with control, non-replicating versions.

We next examined the role of RNA1 3′ end sequence in replication efficiency. A 9-nucleotide insertion directly downstream of ORF A stop codon is sufficient to reduce replication levels of Noda-bsiw 2–3 fold (Fig. 2B). It is well established that the 3′ end of nodaviral RNA1 forms a structure termed 3′ Replication Element (3′RE), which is required for RNA1 replication [37], [38]. However, this structure has not been characterized. Our data shows that the 3′RE likely extends upstream into ORF A, and that splitting the ORF-encoded (blue bars in Fig. 4A) and 3′UTR-encoded (red bars in Fig. 4A) parts of 3′RE lowers replication efficiency.

We used MFold [39] to examine potential secondary structures which can form at the 3′ end of RNA1. MFold predicts a 13-base pair stem-loop within the ORF, directly adjacent to the stop codon (“ORF stem-loop”, Fig. 4A). It also indicates the presence of RNA secondary structures in the 3′ UTR, such as the 3′UTR stem-loop I (Fig. 4A). Thus, 3′RE may consist of 3′UTR and ORF-encoded modules.

Since our data predicts a proximity-dependent interaction between such modules, we wondered whether restoring the ORF-encoded module to the 3′ end of the replicon could reestablish efficient replication. Therefore, we systematically introduced different length fragments of RNA1 derived from the 3′end of the ORF back into Pol-2A-Luc 3′UTR (Fig. 4A, Noda-Pol2A-Luc(30) or -Luc(75), or -Luc(140) constructs). This design leads to the duplication of the 3′RE ORF module: the 5′ repeat at the end of ORF A is translated, while the 3′ repeat (hatched box) is not, as it is downstream of the luciferase stop codon. We found that 75- and 140-nt inserts increase Luc expression approximately 6-fold with respect to control; in contrast, a 30-nt long insert was not sufficient (Fig. 4C). Therefore, the 75 nucleotide genomic segment likely forms an RNA structure that needs to adjoin the 3′ UTR of RNA1. Notably, the 75-nt segment encompasses the predicted ORF stem-loop, while the 30-nt segment does not.

This RNA segment could act at either the level of mature RNA1 (increase translation, replication, or stability of the replicon RNA), or during the production or processing of the initial RNA1 transcript from the pNodaPol2A plasmid (i.e. increasing transcription or nucleocytoplasmic transport). To distinguish between these possibilities, we produced Pol-2A-Luc replicons by in vitro transcription using T7 RNA polymerase, and electroporated them into BSR cells (Fig. 4D). Luciferase measurements showed that replicons containing the 75 nt-long RNA segment exhibited higher Luc activity than replicons missing these sequences. Interestingly, the GAA polymerase mutant replicon also expressed significantly higher levels of luciferase at early time points if it contained the 75-nt extension. This observation suggests that this RNA element, positioned next to the translational stop of the A and B2 ORFs, may enhance translation or stability of the viral RNA (see Discussion).

### Functional replicons with a very unusual protein A C-terminal region

**Figure 5:**
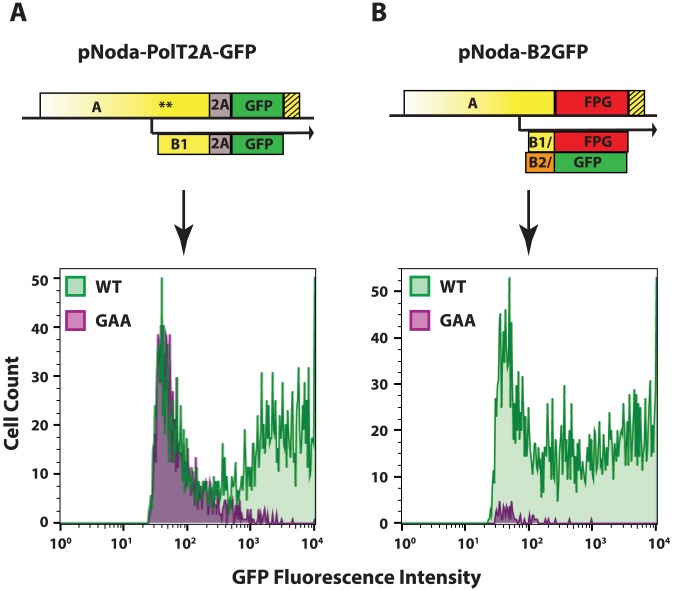
GFP expression from the two replicon designs. **A.** Protein A fusion replicon construct and FACS analysis, **B.** Protein B2 fusion replicon construct and FACS analysis. The hatched yellow box represents the extension of the RNA element, as in Fig. 3. 48 hours after transfection into BSR, cells were trypsinized and examined by FACS. Cells in the GFP-positive gate are shown. Cells transfected with wt polymerase constructs are shown in green; those transfected with GAA mutant polymerase constructs are shown in purple.

The Nodavirus replicons described here efficiently express a transgenic GFP reporter. These modifications included incorporation of the ORF RNA element at the 3′end of RNA1 and an optimized choice of an efficient 2A cleavage site. Median fluorescence intensity of GFP in replicon-transfected cells was found to be 17.5 times higher than in cells transfected with the non-replicative GAA control, due to the presence of a population of very brightly fluorescent cells (Fig. 5A, pNoda-PolT2A-GFP).

Flock House Virus (FHV), a close relative of NoV, has also been engineered as a replicon using a different strategy. In this case, the bulk of ORF B2 was replaced with GFP [40]. In examining the design of that original FHV-GFP construct, we found that protein A was not truncated at the point where the abbreviated protein B2 ORF was fused to GFP. Instead, the overlapping protein A ORF continued uninterrupted throughout the GFP ORF in an alternative reading frame.

Given that the FHV replicon could replicate successfully, we constructed a similar NoV replicon, pNoda-B2GFP, with GFP ORF fused to Glu24 of B2. In this construct, the C-terminal 114 amino acids of B2 were replaced with 239 amino acids of GFP. This replacement in ORF B2 resulted in a concomitant replacement of the protein A C-terminus sequence, where the C-terminal 112 amino acids in ORF A were replaced with a completely different 250 amino acid sequence which we termed “FPG” (Frameshifted Protein derived from GFP) (see Fig. 5 for a scheme of pNoda-B2GFP and Fig. S2 in S1 Text for the sequence relationships between different ORFs). This construct, pNoda-B2GFP, replicated efficiently, judging by the very high levels of GFP expression (Fig. 5B, lower panel). Furthermore, the GFP background in non-replicative GAA replicon is greatly reduced because the transgenic GFP reporter is only produced by the replicated subgenomic RNA3 (Fig. 5B).

### FPG shares some biophysical characteristics with the C-terminus of NoV protein A

We were intrigued by the unexpected ability of FPG – a completely different protein sequence with no previously known functions – to functionally substitute for the native C-terminus of protein A. The C-terminus of protein A and FPG share no significant sequence similarity (as determined by a BLAST comparison [41]), and there are no shorter segments that can result in a significant alignment between the two (as determined independently using the alignment program MUSCLE [42]).

**Figure 6:**
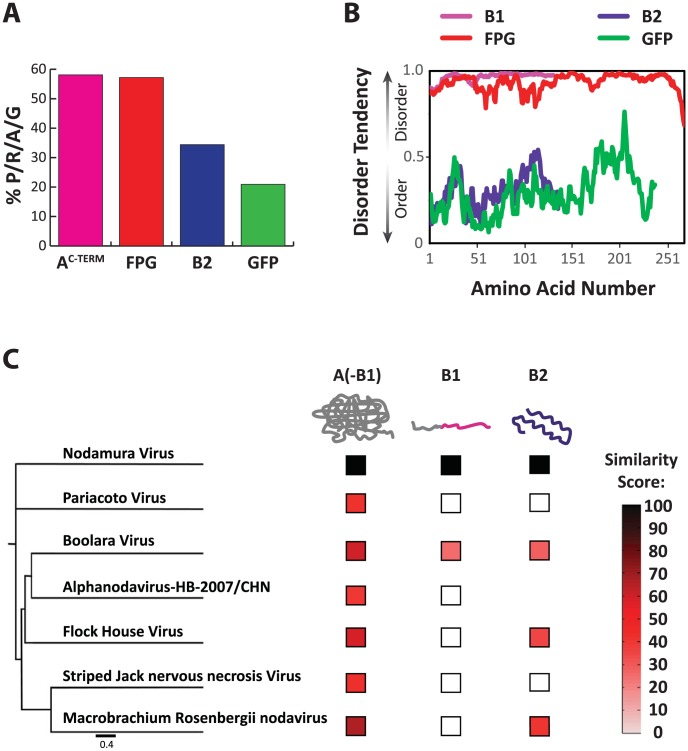
Characteristics of the A^C-TERM^ region and related sequences. **A.** Percent of the amino acids proline, arginine, alanine and glycine (P/R/A/G) in the total amino acid composition of each shown protein. **B.** Disorder tendency plotted for B1, FPG, B2 and GFP. Values above 0.5 indicate disorder, values below 0.5 indicate structured regions. **C.** Similarity of each of the three nodaviral family protein regions to the corresponding ortholog of NoV. The protein regions are as follows: A(-B1): N-terminal of A up to the B1 ORF; B1; and B2. The nodavirus family tree is based on the capsid protein sequences. Similarity score was calculated by multiplying percent similarity between aligning regions of two proteins by the length of the similarity region, and dividing by the total protein length.

We compared the amino acid composition of the wildtype C-terminus of NoV protein A with that of NoV protein B2, FPG, and GFP (Fig. 6A), and found that four amino acids are significantly enriched in both A ^C-TERM^ and FPG. Indeed, the combined percentage of prolines, arginines, alanines and glycines (% P/R/A/G) reached almost 60% in both polypeptides. This was in stark contrast to B2 and GFP, where the P/R/A/G proportion was significantly lower (Fig. 6A). Three of these amino acids (proline, arginine, glycine) are known to be enriched in disordered regions in polypeptides [18], [43]–[46]. Indeed, when analyzing the profile of these two proteins, we observe that unlike structured proteins such as GFP and B2, both nodavirus A^C-TERM^ and FPG are predicted to be intrinsically disordered – that is, their sequences are not expected to fold into a 3-dimensional structure (Fig. 6B). Another striking feature of both sequences is their relatively high net positive charge (+14 in the 112 amino acids of A^C-TERM^ and +45 in the 250 amino acids of FPG). We speculate that some of these features, which are serendipitously shared by the two unrelated sequences, mediate the essential function/s required for RNA1 replication.

### Diversification of the disordered protein A C-terminus, A^C-TERM^

When two viral ORFs overlap, often at least one of them encodes a disordered polypeptide region within the overlapping frame [47]. When we analyzed the A^C-TERM^ region of various other nodaviruses, we observed that, while they have low sequence conservation and different chain lengths, they are all predicted to be highly disordered (using IUpred [48]). The overlapping frame's protein B2 is known to fold into a simple structure [49]–[51]. B2 is more conserved than A^C-TERM^ (Fig. 6C), consistent with the idea that disordered regions tend to evolve faster than structured regions [27], [29], [52]. Thus, while B2 is a structured and relatively conserved protein, A^C-TERM^ is highly disordered and poorly conserved, to the point that almost no similarity exists among A^C-TERM^ proteins of various Nodaviridiae family members.

### Characterization of amino acid sequence at the A^C-TERM^ required for RNA replication

**Figure 7:**
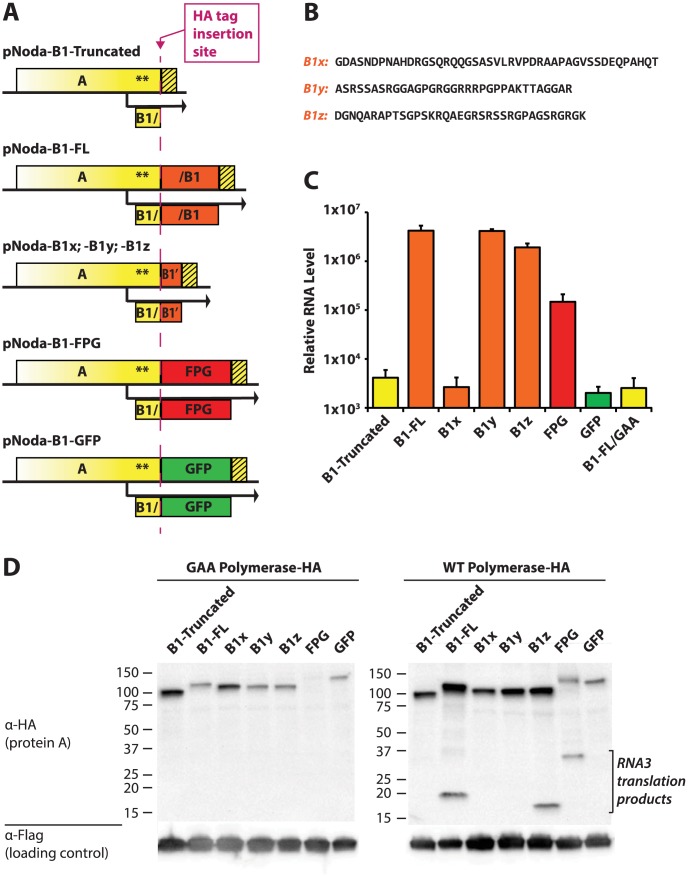
Deletions and substitutions in the A^C-TERM^ region. **A.** Design of various constructs. In the design schemes, yellow color denotes nucleotide sequences derived from RNA1, and orange color corresponds to B1-derived viral sequences which were coded with synonymous codons. HA tag-containing constructs (used in panel D) were constructed by inserting the HA tag in front of the various A^C-TERM^s, as shown by the dashed magenta line. **B.** Amino acid sequences of the three B1 segments, cloned behind the AscI site. **C.** Replication levels of the various constructs were determined by quantitative PCR of RNA1. **D.** Western blot of the HA-containg constructs transfected into BSR cells. Non-replicating (GAA) polymerase constructs are shown on the left; corresponding WT polymerase constructs are on the right. Lower molecular weight bands on the right correspond to RNA3 translation products. The loading control was provided by the plasmid pEF6-GFPflag, which was co-transfected with the Nodamura replicon-expressing plasmids (lower panels).

Our observation that two vastly different sequences, which are both disordered, can support replication, as well as the lack of conservation among A^C-TERM^ sequences across the nodavirus family, led us to further explore the essential features within this region. We created a new set of replicons where the C-terminal region of protein A was replaced by various engineered sequences which lack alternative (protein B2-derived) ORFs (Fig. 7A). All inserts were encoded by nucleotide sequences unrelated to the original viral RNA sequence in order to eliminate any RNA1 structure effects on replication. RNA1 levels were determined by qPCR, as described above.

As expected, deletion of the protein A C-terminus prevented RNA1 replication (Fig. 7C, compare Noda-B1-Truncated with the negative control replicon Noda-B1-FL/GAA). A wt A^C-TERM^ fragment (full-length B1: Noda-B1-FL), however, produced RNA levels 3 orders of magnitude above those of the GAA negative control replicon. We note that A^C-TERM^ is required for RNA1 replication at the protein rather than at the RNA level because replication proceeded despite the fact that wt RNA sequence of A^C-TERM^ was replaced with synonymous codons.

We next substituted A^C-TERM^ with three fragments derived from the A^C-TERM^ sequence (B1x, B1y, B1z, Fig. 7B). Each of these fragments is about 35 amino acids in length; they correspond to the N-terminal, middle and C-terminal thirds of A^C-TERM^, respectively. To our surprise, two of these segments restored replication to wildtype levels (B1y and B1z, Fig. 7C), as we would anticipate that only one, if any, of the 3 segments would incorporate the region needed for replication. This is despite the lack of significant similarity between any of the segments (as determined by BLAST). GFP and its frameshift (FPG) inserts, used in the same experiments, confirm that a mere extension of protein A (by GFP) is insufficient for activity, while the FPG insert stimulates RNA1 replication well above that of truncated or GFP controls (Fig. 7C). Thus, several dissimilar, yet all structurally disordered, sequences can support RNA1 replication.

While A^C-TERM^ is required for amplification of RNA1, its activity may not be influencing the process of RNA replication as such. It is possible that its importance lies in enhancing translation of RNA1 or stability of protein A. We thus further examined the role of A^C-TERM^ by modifying all the replicons in Fig. 7A via addition of the HA tag between the bulk of protein A and A^C-TERM^ (magenta line in Fig. 7A). Protein A expression from HA-containing constructs mirrored RNA replication of non-HA constructs, in a manner dependent on the exact A^C-TERM^ present (Fig. 7D, right panel). However, protein A levels in non-replicative (GAA mutant) HA constructs did not correlate with the replication efficiency of the corresponding replication-proficient replicons (compare the left and the right panels in Fig. 7D). This supports the idea that A^C-TERM^ plays a direct role in RNA replication, rather than in translation or stability of protein A itself.

### The disordered nature of A^C-TERM^ is not sufficient to support replication

**Figure 8:**
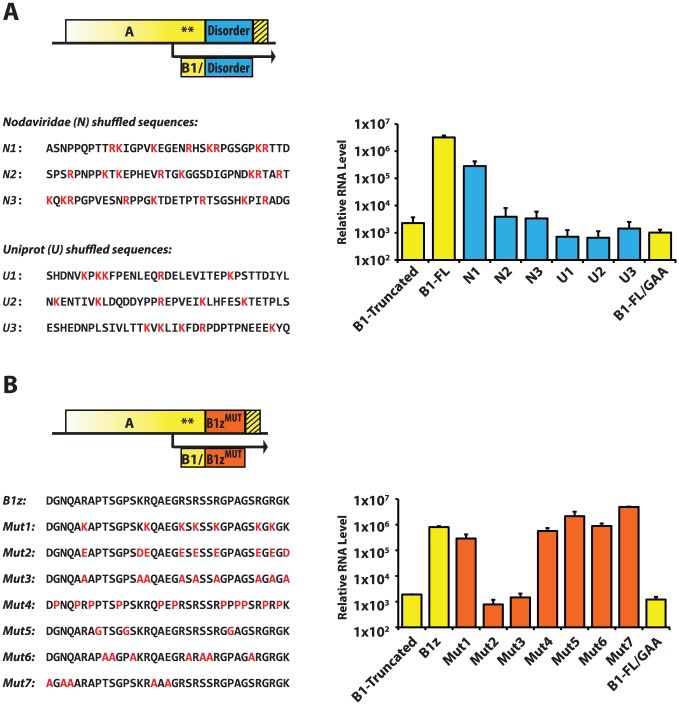
RNA1 replication: Sequence requirements in the A^C-TERM^ region. **A.** Blue box represents the 35-amino acid peptide designed by shuffling of a set of amino acids corresponding to the average (disordered) *Nodaviridae* B1 protein (N1–3), or to the average disordered protein content of the entire Uniprot database (U1–3). The sequences are shown below the scheme; positively charged amino acids are in red. RNA levels were measured by RNA1 quantitative PCR. **B.** Orange box represents various mutants of the 35-amino acid B1z region. Their sequences are shown below. Mutations are in red. qPCR results are shown on the right.

We next examined whether the disordered nature of the A^C-TERM^ sequence, by itself, is sufficient to support replication. To this end, we replaced A^C-TERM^ in the wildtype protein A replicon (pNoda-B1-FL) with artificial 35-amino acid tails. We designed these fragments to approximate a random disordered region, and based them on either the average composition of protein B1 of various nodaviruses (“N sequences”, Fig. 8A), or the total amino acid content of all known disordered regions in the entire Uniprot database [53] (“U sequences”, Fig. 8A). Three different amino acid sequences were cloned for each of these two amino acid compositions (N1, N2, N3 and U1, U2, U3), and in each case the order of residues within the constructs was randomly assigned. Thus, the N sequences contained the same set of amino acids differing between the three variants in their exact positions; and the U sequences contained a different set of amino acids which was similarly shuffled from one U sequence to the next (see table S3 in S1 Text for the relative fractions of each amino acid in these sequences, and Fig. 8A for their precise sequences).

As shown in Fig. 8A, only one of the six constructs – N1 – was active. This suggests that the disordered nature of A^C-TERM^ is likely to be required but is insufficient for the RNA replication activity of protein A, and that certain uncharacterized features within these disordered regions are needed to support replication. In addition, these results exemplify the rapid evolvability of the functions encoded in the disordered C-terminus, as one in six randomly designed disordered regions is indeed able to support replication.

### A specific arrangement of positively charged amino acids is required at A^C-TERM^

To further characterize the features of A^C-TERM^, we performed a systematic mutagenesis of the amino acids in the B1z construct. A given type of amino acid was mutated in a concerted fashion to a different amino acid throughout B1z. Thus, arginines were mutated to lysines (Mutant 1), to glutamates (Mutant 2), or to alanines (Mutant 3); Mutant 2 also contained the lysine-to-aspartate, and Mutant 3 – lysine-to-alanine substitutions to remove all positive charge. In Mutant 4, glycines and alanines were mutated to prolines; in Mutant 5, prolines were mutated to glycines, and, in Mutant 6, serines and threonines were replaced with alanines. Finally, several mutations were built into the tail to remove the few amino acids which are not proline, arginine, alanine, glycine and serine/threonine (the most common residues in the original B1z sequence; Mutant 7). Amino acid sequences and replication levels for all of these constructs are shown in Fig. 8B. Strikingly, all of the mutants, with the exception of Mutant 2 and Mutant 3, in which positive charges were removed, replicated efficiently. Therefore, we conclude that two major features characterize the C-terminus of protein A: structural disorder and the requirement for positive charges. Additionally, it appears that the positively charged amino acids may need to be arrayed in some particular order, since not every sequence carrying the same charge can support replication (Fig. 8A, see constructs N2 and N3).

## Discussion

Nodavirus is a small RNA virus, with only 4,500 bases and 3 ORFs. In this study we discovered two uncharacterized elements within the 3′end of nodavirus RNA1. One of them is an RNA element adjacent to the stop codon of ORF A, and the other is the unstructured C-terminus of the RdRp, which is essential for replication. The C-terminus of the RdRp has unusual properties that provide new insights into the evolutionary plasticity of intrinsically disordered proteins, as well as their utilization by viruses. In addition to illuminating the biology of nodaviruses, these features can be exploited to create new NoV-based replicons.

### 3′ replication element in NoV extends into the ORF

While attempting to explain decreased replication of some initial replicon constructs, we found an RNA element straddling the stop codon of the ORF A. It seems likely that this RNA element is a part of the previously observed 3′ Replication Element of RNA1 (3′RE) in FHV, which was found to cover almost the whole RNA3 region in FHV [38]. Here we find that some portions of this region are in fact dispensable for RNA1 replication, at least in NoV (nucleotides 2816–3078 of NoV RNA1). Our results suggest that the 5′ border of this element probably lies in the coding region between nucleotides 3079 and 3124 of NoV RNA1. Our findings resemble those of Albarino et al [37], who found that the *in trans* replication of FHV RNA1 requires only the 108 3′-most nucleotides in 3′RE – and that this region extends 5′ of the stop codons of the A and B2 ORFs. Of note, our analysis and Albarino′s study used mammalian cells, while the study by Lindenbach et al. was conducted in yeast. RNA1 replication requirements in these systems may be somewhat different.

The exact role of the 75 nucleotides at the 3′ end of the protein A ORF is not yet clear. It seems that the 3′RE can be split into two parts, the 3′UTR module and the 75-nt ORF module (red and blue bars, respectively, in Fig. 4A). We note that RNA1 derivatives with the full 3′RE produce more luciferase upon electroporation into BSR cells than those whose ORF module does not adjoin the 3′UTR module (Fig. 4D), even when these RNA1s encode inactive polymerase. Improved stability can be imparted on nodaviral RNA by protein A [54], and it is possible that the 75-nt RNA region provides an additional binding site for the RDRP. However, it appears that RNA remains relatively stable with or without full 3′RE, as luciferase expression does not decrease appreciably over the first 8 hours. Thus, although our study is not conclusive with respect to the mechanistic role of this RNA structure, it is mostly consistent with the idea that this RNA element ensures efficient translation, rather than improved stability, of RNA1. Additionally, we cannot exclude a direct role for the 75nt module in replication.

*In silico* RNA structure prediction suggests the presence of two separate stem-loops (Fig. 4B), one formed by nucleotides 3113–3142 (the ORF stem-loop), and the other, by nucleotides 3162–3177 (the 3′UTR stem-loop I). However, since efficient replication depends on the proximity of the ORF module and the 3′ UTR module (Figs. 2B and 4C,D), we speculate that these RNA structures may function as a single RNA element. We also note that the loop of the RNA1 3′UTR stem-loop I proposed here (Fig. 4B) contains the same hexanucleotide sequence CCAUCU that forms the loop of the recently characterized 3′SL replication element in NoV RNA2 [55]. This sequence, perched at the end of a stem-loop, may serve as a common binding site for the polymerase, or a site for an RNA-RNA interaction.

### NoV replicons for the efficient expression of foreign genes

A replicon's utility is fully realized in its ability to encode a foreign gene. Currently, several designs have been published which allow one to express a reporter gene in a nodaviral system. Some schemes require the use of two co-transfected plasmids [55], [56]. A simpler scheme requiring only one RNA1-based plasmid would be desirable. Such systems have been established for FHV. In one design (the protein A-fusion), GFP fused to the C-terminus of the FHV polymerase resulted in a functional replicon [32]. In a different design (protein B2-fusion), GFP was used to replace most of the B2 ORF [40]. Here, we describe replicons which can efficiently express transgenes from NoV RNA1 (Fig. 3).

Of the two possible transgene designs, protein A fusions (Fig. 5, panel A) can express any transgene which tolerates an N-terminal proline in mammalian cells. The alternative, protein B2-fusion approach (Fig. 5, panel B), has proven much more surprising. While GFP is expressed comparably from both protein A and protein B2 fusions, the background fluorescence is lower in the latter. This is expected, since the B2-GFP ORF can be translated only from replicating RNA3, but not from the primary unreplicated transcripts. However, protein B2-GFP construct design cannot be extended to most other genes, as it relies on a very particular alternative ORF of GFP, which we named FPG.

In contrast, the replicons described here allow for robust expression of an ectopic protein that is not fused to protein A or to B2. The use of this type of replicons may need to be carefully optimized since NoV lyses infected cells. We wondered if, unlike the wild-type virus, capsid-deficient replicons described here may maintain long-term replication in cells. However, our initial observations indicated that robust replication of these replicons may also lead to cell lysis.

### Structural characteristics of the C-terminus of protein A

Several functional domains have been mapped in protein A. Its central domain contains the catalytic core of the viral polymerase, and its N-terminal third is hypothesized to encode an RNA capping enzyme [5]. The extreme N-terminus of A localizes the protein to mitochondrial membranes [57], [58]; additionally, multimerization motifs are present throughout the protein [59]. However, the function and requirements of its C-terminus (A^C-TERM^) have remained unclear.

The finding that a completely unrelated protein sequence, FPG, can replace the original A^C-TERM^, was quite unexpected. We hypothesized that its disordered nature, and/or amino acid composition, may mimic the features of the natural A^C-TERM^. Strikingly, A^C-TERM^ could be shortened to less than one third of its size (35 amino acids) without losing function. Furthermore, either the middle third or the C-terminal third of the sequence could fulfill the requirements for RNA1 replication (Fig. 7) despite the lack of any significant similarity between the two sequences. We found that even a randomly shuffled disordered sequence could support the replication function of A^C-TERM^ (Fig. 8A) – a unique situation rarely encountered in proteins. However, other disordered sequences with similar compositions had no activity (Figs. 7B and 8), indicating that a pattern, and not merely a set, of amino acids was required for function. Further examination revealed that positive charges, preferably arginines, but not other specific amino acids, are necessary for the function of A^C-TERM^ (Fig. 8B). Thus, successful RNA1 replication requires a pattern of positive charges in the context of a disordered region.

Positively charged amino acids, and arginines in particular, are known to facilitate binding to RNA [60], [61]; thus the C-terminus of protein A may be required for interactions with viral RNA. Interestingly, intrinsically disordered regions are thought to be important in regulation of nucleic-acid binding [22], [23], [62]. Alternatively, A^C-TERM^ could function much like a positively charged amino acid cluster in FHV coat protein, known to direct the protein to mitochondria [63]. It is noteworthy that membrane localization of protein A is not entirely dependent on the N-terminus [57], [58]; therefore, a contribution from the C-terminus may also be required for subcellular localization of the replication complex.

Our evolutionary analysis (Fig. 4) demonstrates that an exceptionally diverse array of sequences serve as C-termini for the polymerases of the *Nodaviridae*. This analysis strongly supports the concept that the sequence requirements placed on the C-terminus of protein A are remarkably relaxed. It is important to note that, while we demonstrate a very limited set of requirements imposed on A^C-TERM^ itself, there are other constraints on its sequence. Since ORF B2 overlaps ORF A, there must be a strict limit to the diversity allowed at A^C-TERM^. Indeed, it has been shown that in general, overlapping reading frames tend to be relatively conserved [64]. Furthermore, as we show above, the region encoding the last 20 or so amino acids in A^C-TERM^ contains an RNA element important for RNA amplification. In light of this, it is especially remarkable that A^C-TERM^ is the most variable protein region in the virus (Fig. 6C). In part, this may be due to the fact that B2 can also accommodate a lot of variation (Fig. 6C). We propose that because A^C-TERM^ is disordered, it can tolerate higher sequence diversity without compromising its overall structure or stability. As a consequence, the disordered region may allow for a more thorough sequence space exploration, which may result in the generation of novel, adaptive functions (see below). Taken together, our analysis indicates that the extreme sequence plasticity of the C-terminus of Protein A has been exploited during evolution, leading to the diversification of protein sequence in this region.

### Disordered regions and the maintenance of protein function during virus evolution

In this study, we integrated two different and complementing analyses. First, by using structure predictions and evolutionary comparisons, we demonstrated that protein A C-terminus is highly disordered and that its similarity across different Nodaviruses is extremely low in comparison to other nodaviral proteins. Second, we showed that replacing the original C-terminus sequence with other disordered regions – some with no apparent similarity – still allows replication. We thus discovered that a rather extended protein region, which is required for a core viral activity exhibits a remarkable robustness of its function in the face of numerous and drastic sequence changes.

Many studies have presented extensive computational analyses of disordered proteins and their evolution [28], [29], [65]. They are in broad agreement that disordered protein regions evolve rapidly; however, the exact extent to which these regions can be mutated and yet maintain their function has not been explored. Here, we have marshalled both computational and experimental evidence to show a remarkable capacity of structurally disordered regions to evolve fast while maintaining essential functions such as virus replication.

### Disordered regions and the functional diversification of viral proteins during evolution

Intrinsically disordered protein regions are now appreciated as an extremely versatile part of the proteome. Their conformational flexibility and potential for mediating multiple intermolecular interactions is thought to allow them to rapidly acquire novel functions in the course of evolution. Comparative studies of orthologous sequences in various eukaryotes have revealed that disordered regions indeed evolve exceptionally fast [27], [28]. NoV A^C-TERM^ represents a notable illustration of this rapid evolution.

Viral genome size is constrained. In principle any “extra” genomic sequence space in a virus would be lost without a selective pressure to maintain it, yet a rather extended A^C-TERM^ persists throughout Nodaviridae. While it is possible that maintaining the entire length of this segment is selectively neutral, we hypothesize that mechanisms to maintain it can exist and provide a selective advantage. Recent studies suggest that viruses exploit short peptide motifs residing in disordered regions to evolve many of their interactions with host proteins [12], [13]. Here, we demonstrate that the disordered C-terminus of NoV protein A, which is essential for viral RNA1 replication, exhibits extremely lax sequence requirements for function, and thus has the potential to incorporate new motifs and functions during evolution. From the point of view of viral evolution, then, the relaxed nature of the A^C-TERM^ sequence effectively allows the diversification of the sequence in this region without a significant loss of fitness. This in turn may provide a rich reservoir of novel sequences and functions. Additionally, the variability of the A^C-TERM^ sequence can be the result of neutral selection where replication function is maintained and the rest of the sequence changes under no selective pressure. At the moment we do not know whether or not the A^C-TERM^ sequence indeed carries out additional functions. Future experimentation will be necessary to determine the specific role of this region in a given host or environment.

In this regard, it is notable that protein B1, which is colinear with A^C-TERM^, is expressed from RNA3 [6]. B1 expression is dispensable for RNA replication [6], but B1 may harbor additional functions. Whereas a limited number of amino acids are required for RNA replication at A^C-TERM^, a different subset of amino acids in this region can fulfill a different function as a part of a (free) B1 protein or as the C-terminus of protein A. This sequence and functional flexibility underscores the remarkable fact that this region of the viral genome can maintain function through the conservation of a few dispersed residues while allowing evolution to produce highly diversified intervening sequences.

## Materials and Methods

### Plasmid construction

Freeze-dried Nodamura virus, strain Mag115, was obtained from ATCC (Cat. No. VR-679) and resuspended in 1 ml of water. 250 ul of this suspension was processed with 750 ul of TRIzol LS according to the manufacturer's protocol. Following cDNA synthesis using random hexamers and SuperScriptIII (Invitrogen), several primer pairs were used to amplify, TA-clone and sequence the amplicons (noV1Fpst2: 5′-ccac ctgca gtattgaatccaaaactcaaaatgctgaac-3′ with noda1654R: 5′-GAT CAC GGA ATG CCA GCG TAT AGC TGG AAA ACC G-3′; noda1383F: 5′-caaggtccactggccagcgcacgtcgaag-3′ with NoV1-RT: 5′-ACC ACT GGC ATA AGC CTA GTT C-3′ were used for the initial cloning and sequencing. For amplifying the 5′-3′ end junctions, primers nov1-2938F (5′-catcaaaccgcgagtcgcag-3′) and nov1-171R (5′-CGT GCG TCG ATG CAC GAT-3′) were used). 5′ genomic and 3′ genomic clones were then merged and cloned behind the SV40 minimal early promoter (amplified with sv40promFsac: 5′-ccac ttataa gcgatcgc gagctc tgcatctcaattagtcagcaacc-3′ and sv40pRpst: 5′-CTGCAG CGG CCT CGG CCT-3′). Standard cloning techniques were employed for construction of these and other plasmids; PCR was conducted with Phusion proofreading polymerase (New England Biolabs).

Protein A expression plasmids did not contain 5′ and 3′ UTRs of RNA1; furthermore, a number of synonymous mutations were introduced into the 3′ third of protein A ORF (by gene synthesis: BioBasic, Inc.) in order to remove any RNA replication elements. GFP used in our replicons is a brighter variant of GFP with a very similar nucleotide sequence, called Venus [66].

Plasmid sequences and details of construction are available upon request.

### Cell culture and replication analysis

BSR cells (which do not express T7 polymerase) were obtained from the Matthias Schnell laboratory and cultured in the DMEM-high glucose medium (UCSF Cell Culture Facility) supplemented with 10% FBS (Sigma), glutamine, penicillin-streptomycin and non-essential amino acids (UCSF CCF). Transfections of the NoV replicon-expressing plasmids were done in 24-well plates (using 20–50% confluent cells) with 1 ul of LipofectAMINE2000 and 0.5 ug of plasmid(s) according to the manufacturer's protocol.

For qPCRs, RNA was subsequently collected using 0.3 ml Trizol (Invitrogen) per well, precipitated and resuspended in 25–60 ul of water. 1.5–2.5 ug total RNA was treated with DNaseI (Promega) and, following DNase inactivation at 75°C, 200–500 ng was used in a SuperScriptIII (Invitrogen) reverse transcription. First strand cDNA was used in the qPCR run with the SYBR FAST Universal 2× qPCR Master Mix (Kapa Biosciences) on a CFX-Connect cycler (Biorad). Primers used in the PCR were: noda1219F (5′-gccataaatcccaaggtccactg -3′) and noda1325R (5′-GGC ATC ATA TTT TCG TCA GAT ACC AAC G -3′) to amplify NoV RNA1, hamsteRplF (5′-AGC CCG TGA CTG TCC ATT C -3′) and hamsteRplR (5′-GGC AGT ACC CTT CCG CT -3′) to amplify the hamster Rpl19 message, in a final volume of 10 ul. Cycling conditions were: 95°C for 10 seconds, 62°C for 20 seconds, and 72°C for 30 seconds, for 40 cycles. 1∶5 or 1∶200 dilutions of the cDNAs were used as the starting material. “No RT” controls were always run in parallel to ensure that the signal did not originate from the DNase-undigested plasmid. Relative amount of NoV replicons was obtained by referencing the Noda signal to the Rpl19 signal (delta-delta Ct method).

For in vitro transcriptions, SV40 promoter was substituted with the T7 promoter in the relevant plasmids, and the plasmids were linearized at the XbaI restriction site 3′ of the HDV ribozyme. Transcriptions were conducted with mMessage mMachine reagents from Ambion, as suggested by the manufacturer, using the cap analog∶GTP ratio of 4∶1. Northern blotting was done according to the standard protcols [67], after running RNA in formaldehyde gels and transfer to nitrocellulose membranes. The Northern probe was labeled by random priming of the PCR product, the 3′-most 1167 nucleotides of RNA1, derived by amplifying pNodBall (see text) with noda2038F (5′-caatattgctccattcgaatgacacacccagagc-3′) and NoV1-RT. Hybridization at 42°C in UltraHyb buffer (Ambion) was followed by washes at 50 and 55°C in 2×SSC/0.1% SDS and 0.2×SSC/0.1% SDS, respectively.

Dual Firefly/Renilla luciferase assays were run using Promega's Dual Luciferase Assay system according to the manufacturer's protocol. Cells were lysed in 50 ul of Passive Cell Lysis buffer per one well of the 24-well plate. 10 ul of the cleared lysate was added to 70 ul of the Firefly assay reagent, followed by 70 ul of the Renilla assay reagent. Measurements were conducted in Tecan's UltraEvolution 96-well plate luminescence reader. Similar protocol was followed when using single firefly, Luciferase Assay System (Promega).

For flow cytometry, transfected cells were trypsinized, washed and resuspended in PBS with 2% FBS, then analyzed on FACScalibur (Becton-Dickinson) for GFP expression; FlowJo software was used for data processing.

For Western analysis of 2A-mediated cleavage, cells were lysed 48 h after transfection of 30%-confluent BSR in a well of a 6-well plate, in 75 ul of cytoplasmic lysis buffer (150 mM KCl, 2 mM MgCl_2_, 30 mM Hepes pH 7.4, 0.5% NP40, protease inhibitors (Roche)). 20 ul of the resulting sample was run in a 4–12% NuPAGE denaturing polyacrylamide gel (Invitrogen). Samples were then transferred onto a PVDF membrane and probed with an anti-GFP rabbit polyclonal (sc-8334, Santa Cruz Biotechnology) and an HRP-conjugated donkey anti-rabbit IgG-F(ab)_2_ (GE Healthcare), both at 1∶2,500. ECL system (Pierce) was used for signal detection.

Western blot of the HA expression replicons was done by lysing BSR cells 24 hours after transfection (as above) with a mixture of the replicon (1.5 ug) and pEF6-GFPflag internal normalization control plasmid. 40 ul of the RIPA buffer with protease inhibitors (Roche) was applied per well of a 12-well plate. 10 ul of each sample was loaded per lane of a 4–20% Biorad denaturing polyacrylamide gel. After transfer to a PVDF membrane, anti-HA mouse monoclonal (6E2, Cell Signaling #2367) was used at 1∶1000, or anti-Flag mouse monoclonal M2 (F3165, Sigma) was used at 1∶25,000. Secondary antibody (sheep anti-mouse HRP-conjugated IgG-F(ab)_2_) was bought from GE Healthcare (NA9310V) and used at 1∶2,500 to detect 6E2 and at 1∶10,000 to detect M2. Signal was developed using the ECL system (Pierce).

### RNA element analysis

For RNA structure predictions, we accessed an MFold server (http://mfold.rna.albany.edu/?q=mfold/RNA-Folding-Form) and submitted queries using the Web interface. Standard conditions were chosen, and 5 resulting structures were examined.

### Nodaviridiae family and protein analysis

We assembled a set of 7 viruses belonging to the alpha and beta branches of the nodavirus family. For each of the 7 viruses, we collected the capsid polyprotein, the RDRP (protein A), and protein B2 from the NCBI online database (see links in table S2 in S1 Text). We inferred the sequence of protein B1 – the C-terminus tail of protein A, when it was not available online, based on sequence similarity to other family members or based on the existence of a methionine which presumably acts as its initiation site. Using BLAST [41], we calculated the similarity score of each of the protein (A,B1,B2) with respect to the nodavirus orthologs, by finding the region that is most significantly similar to the nodavirus protein in the orthologous protein, and computing a normalized similarity score as: (the length of the similar region)×(% similar residues that are similar in the alignment in this region)/(total length of the orthologous protein). The results of the normalized similarity scores for protein A, B1 and B2 appear in Fig. 4, with a phylogenetic tree that was constructed based on the capsid proteins. The tree was formed by creating an alignment using the MUSCLE program [42] and by using the PhyML program with default parameters [68]. The tree figure was created using the FigTree program (http://tree.bio.ed.ac.uk/software/figtree/).

We predicted the disorder profile of each of the protein A's sequences using the disorder predictor IUpred [48], using the default parameters and the ‘long’ version (which is optimized to search for long stretches of disordered regions, such as the C-terminus tail of protein A). IUpred was shown to give similar predictions to other methods and to be in a strong agreement with experimental data [69]. A residue was considered to be ‘disordered’ if its predicted disorder value was 0.4 or higher (in the scale of 0 to 1). We then computed the average AA content of the 7 B1 proteins, so that each of the 20 AAs has a fraction of occurrence in the set of B1 proteins (e.g. – on average, 6.3% of the residues in the 7 B1 proteins are lysine residues). See Table S3 in Text S1 for the composition of disordered regions in the *Nodaviridae* B1 proteins.

We downloaded the entire uniprot dataset [53] (version 2013_1), and predicted the disordered regions of each of the proteins as described above, and calculated the average disordered content of the entire proteome (using all the residues in the uniprot dataset that are predicted to be disordered; e,g.– on average, 6.5% of the residues in disordered regions of the entire uniprot set are Lysine residues). See Table S3 in Text S1 for the composition of disordered regions in the entire uniprot database.

We predicted the disorder profile of GFP and its frame-shifted version (“FPG”) using the same parameters. Using BLAST, we searched for sequence similarity between “FPG” and the nodavirus protein A.

Using the fraction of occurrence of residues in disordered regions in the nodavirus B1 proteins, we created three constructs with this amino acid content ordered at random. In each of these constructs we replaced the nodavirus B1 protein with a sequence of 35 residues, that the order of the residues was determined by random using the uShuffle program [70], and the propensity of each residue was similar to its fraction in the B1 proteins. Similarly, we created a set of three 35-AAs long constructs, with amino acid propensities based on the disordered content of the entire uniprot database, with a random order along the construct. The engineered region of each of these constructs sequences was predicted to be disordered according to the procedure described above.

## Supporting Information

Text S1 **Derivation of cDNA for Nodamura virus RNA1 (protocol). Fig. S1. In vivo assay to test the influence of various 2A peptide fusions to the polymerase C-terminus.** **A.** Construct Design. Mutant polymerase-encoding replicon NodaF2A-Luc/GAA was co-transfected with a plasmid which supplied a polymerase in trans from the EF1a promoter. The polymerase-expressing plasmid had all non-coding RNA1 sequences removed and the last third of the RDRP ORF coded with synonymous codons; the synonymous region is depicted in grey stripes. Each transfection also contained a renilla luciferase-expressing plasmid as a normalization control (not shown). Firefly luciferase counts from the pEF6-PolWT transfection are taken as 100%, while polymerases bearing various 2A versions are shown as % of wt value. **B.** Co-translational “cleavage” efficiency of “T2A” and “F2A” peptides in replicons (NodaT2A-GFP, NodaT2A-GFP/GAA) and expression plasmids (pEF6-PolT2A-GFP and pEF6-PolF2A-GFP). The Western blot was probed for GFP. pEF6-GFP is used as a positive control; (-) denotes a lane with lysate from untransfected cells. Arrow indicates the unprocessed PolF2A-GFP polypeptide. **Fig. S2. Sequence of the pNoda-B2GFP replicon in the B1/B2 region.** Wildtype sequence of RNA1 is maintained 5′ of the AscI site (in grey); accordingly, B2 ORF is outlined in purple and A/B1 ORF is in yellow. Insertion of GFP (green, in frame with B2) leads to the replacement of the wildtype ORF A 3′ end with the de novo produced FPG ORF (red). **Table S1. Differences between Mag115 strain sequence and the reference (passaged) sequence. Table S2: Links to viral sequences used in this study. Table S3: Relative composition (% of each amino acid) in disordered regions.**
(DOCX)
