# Supplementary material for: Rapid Evolution of Virus Sequences in Intrinsically Disordered Protein Regions
Source: PLoS Pathog. 2014 Dec 11;10(12):e1004529. doi: 10.1371/journal.ppat.1004529 (PMC4263755; doi:10.1371/journal.ppat.1004529)
Supplement: Text S1 — Derivation of cDNA for Nodamura virus RNA1 (protocol). Fig. S1. In vivo assay to test the influence of various 2A peptide fusions to the polymerase C-terminus. A. Construct Design. Mutant polymerase-encoding replicon NodaF2A-Luc/GAA was co-transfected with a plasmid which supplied a polymerase in trans from the EF1a promoter. The polymerase-expressing plasmid had all non-coding RNA1 sequences removed and the last third of the RDRP ORF coded with synonymous codons; the synonymous region is depicted in grey stripes. Each transfection also contained a renilla luciferase-expressing plasmid as a normalization control (not shown). Firefly luciferase counts from the pEF6-PolWT transfection are taken as 100%, while polymerases bearing various 2A versions are shown as % of wt value. B. Co-translational “cleavage” efficiency of “T2A” and “F2A” peptides in replicons (NodaT2A-GFP, NodaT2A-GFP/GAA) and expression plasmids (pEF6-PolT2A-GFP and pEF6-PolF2A-GFP). The Western blot was probed for GFP. pEF6-GFP is used as a positive control; (-) denotes a lane with lysate from untransfected cells. Arrow indicates the unprocessed PolF2A-GFP polypeptide. Fig. S2. Sequence of the pNoda-B2GFP replicon in the B1/B2 region. Wildtype sequence of RNA1 is maintained 5′ of the AscI site (in grey); accordingly, B2 ORF is outlined in purple and A/B1 ORF is in yellow. Insertion of GFP (green, in frame with B2) leads to the replacement of the wildtype ORF A 3′ end with the de novo produced FPG ORF (red). Table S1. Differences between Mag115 strain sequence and the reference (passaged) sequence. Table S2: Links to viral sequences used in this study. Table S3: Relative composition (% of each amino acid) in disordered regions. (DOCX) [file ppat.1004529.s001.docx]

**SUPPORTING INFORMATION**

Derivation of cDNA for Nodamura virus RNA1

To create NoV RNA1 replicons, we obtained freeze-dried virus from ATCC. Viral RNA, isolated directly from the vial, was used in a random hexamer-primed RT reaction, and then in several PCR reactions to produce a series of amplicons which covered the whole length of RNA1 and RNA2. Since PCR primers had to be placed at the ends of virus genome segments, the sequence of the extreme 5’ and 3’ ends of RNA1 and RNA2 could not be verified initially. As our goal was to study RNA1, we obtained the extreme 5’ and 3’ sequence of RNA1 by transfecting viral RNA into BSR cells and amplifying the fragments across the junctions between head-to-tail RNA1 dimers, which form during nodavirus replication [[1](#_ENREF_1),[2](#_ENREF_2)]. In these experiments, we discovered four differences between the published reference sequence and our clones, summarized in Table S1. A slightly different RNA2 sequence had previously been reported [[3](#_ENREF_3)]. Our sequence of RNA2 is consistent with the cDNA clone of Johnson et al., 2003, except at position 328 (see Table S1).

In the original report describing NoV infectious clone production, it was noted that the mosquito-derived ATCC virus was passaged in suckling mice and then *in G. mellonella* larvae [[2](#_ENREF_2)]. This passaging history could explain the sequence discrepancies observed between the original ATCC virus and the infectious clones derived previously. In order to be able to compare our results to those in the literature, and to examine the possibility that some mutations from the mouse-passaged virus can be adaptive for mammalian cells, we introduced the three RNA1 mutations found in the originally reported infectious cDNA into our mammalian expression plasmid, called pNodBall (Fig. 1). We did not find a significant difference between the level of replicating RNA launched from pNodaRNA1 and pNodBall (not shown), and all subsequent constructs were based on the pNodBall RNA sequence.

pNodBall was designed to express RNA1 from an enhancer-less SV40 promoter [[4](#_ENREF_4)]. Cloning was conducted such that the transcription start site would coincide with the first nucleotide of RNA1, forming a precise 5’ end of RNA. Since a correct 3’ end is also important for NoV RNA replication [[5](#_ENREF_5)], we introduced the genomic ribozyme from the hepatitis delta virus (HDV) [[6](#_ENREF_6)] directly following RNA1 (Fig. 1B). RNA cleavage would then remove the ribozyme and any downstream sequences, freeing the 3’ end of RNA1.

**Fig. S1. In vivo assay to test the influence of various 2A peptide fusions to the polymerase C-terminus.** **A.** Construct Design. Mutant polymerase-encoding replicon NodaF2A-Luc/GAA was co-transfected with a plasmid which supplied a polymerase in trans from the EF1a promoter. The polymerase-expressing plasmid had all non-coding RNA1 sequences removed and the last third of the RDRP ORF coded with synonymous codons; the synonymous region is depicted in grey stripes. Each transfection also contained a renilla luciferase-expressing plasmid as a normalization control (not shown). Firefly luciferase counts from the pEF6-PolWT transfection are taken as 100%, while polymerases bearing various 2A versions are shown as % of wt value. **B.** Co-translational “cleavage” efficiency of “T2A” and “F2A” peptides in replicons (NodaT2A-GFP, NodaT2A-GFP/GAA) and expression plasmids (pEF6-PolT2A-GFP and pEF6-PolF2A-GFP). The Western blot was probed for GFP. pEF6-GFP is used as a positive control; (-) denotes a lane with lysate from untransfected cells. Arrow indicates the unprocessed PolF2A-GFP polypeptide.

**Fig. S2. Sequence of the pNoda-B2GFP replicon in the B1/B2 region.**

Wildtype sequence of RNA1 is maintained 5’ of the AscI site (in grey); accordingly, B2 ORF is outlined in purple and A/B1 ORF is in yellow. Insertion of GFP (green, in frame with B2) leads to the replacement of the wildtype ORF A 3’ end with the de novo produced FPG ORF (red).

**M R L R A N Q V T S S Q A G A**

**atgacaaacatgtcatgcgcttacgagctaatcaagtcacttccagccaagctggagcag**

**M T N M S C A Y E L I K S L P A K L E Q**

**A G S G G R A N G E Q G R G A V H R G G**

**ctggctcaggagggcgcgccaatggtgagcaagggcgaggagctgttcaccggggtggtg**

**L A Q E G A P M V S K G E E L F T G V V**

**A H P G R A G R R R K R P Q V Q R V R R**

**cccatcctggtcgagctggacggcgacgtaaacggccacaagttcagcgtgtccggcgag**

**P I L V E L D G D V N G H K F S V S G E**

**G R G R C H L R Q A D P E A D L H H R Q**

**ggcgagggcgatgccacctacggcaagctgaccctgaagctgatctgcaccaccggcaag**

**G E G D A T Y G K L T L K L I C T T G K**

**A A R A L A H P R D H P G L R P P V L R**

**ctgcccgtgccctggcccaccctcgtgaccaccctgggctacggcctccagtgcttcgcc**

**L P V P W P T L V T T L G Y G L Q C F A**

**P L P R P H E A A R L L Q V R H A R R L**

**cgctaccccgaccacatgaagcagcacgacttcttcaagtccgccatgcccgaaggctac**

**R Y P D H M K Q H D F F K S A M P E G Y**

**R P G A H H L L Q G R R Q L Q D P R R G**

**gtccaggagcgcaccatcttcttcaaggacgacggcaactacaagacccgcgccgaggtg**

**V Q E R T I F F K D D G N Y K T R A E V**

**E V R G R H P G E P H R A E G H R L Q G**

**aagttcgagggcgacaccctggtgaaccgcatcgagctgaagggcatcgacttcaaggag**

**K F E G D T L V N R I E L K G I D F K E**

**G R Q H P G A Q A G V Q L Q Q P Q R L Y**

**gacggcaacatcctggggcacaagctggagtacaactacaacagccacaacgtctatatc**

**D G N I L G H K L E Y N Y N S H N V Y I**

**H R R Q A E E R H Q G Q L Q D P P Q H R**

**accgccgacaagcagaagaacggcatcaaggccaacttcaagatccgccacaacatcgag**

**T A D K Q K N G I K A N F K I R H N I E**

**G R R R A A R R P L P A E H P H R R R P**

**gacggcggcgtgcagctcgccgaccactaccagcagaacacccccatcggcgacggcccc**

**D G G V Q L A D H Y Q Q N T P I G D G P**

**R A A A R Q P L P E L P V R P E Q R P Q**

**gtgctgctgcccgacaaccactacctgagctaccagtccgccctgagcaaagaccccaac**

**V L L P D N H Y L S Y Q S A L S K D P N**

**R E A R S H G P A G V R D R R R D H S R**

**gagaagcgcgatcacatggtcctgctggagttcgtgaccgccgccgggatcactctcggc**

**E K R D H M V L L E F V T A A G I T L G**

**H G R A L Q V K H R Y R R V -**

**atggacgagctttacaagtaaagcatcgataccgtcgagtctag**

**M D E L Y K -**

**Table S1. Differences between Mag115 strain sequence and the reference (passaged) sequence**

| **Segment** | **Nucleotide position** | **Mag115 Nucleotide** | **Mag115 Amino Acid** | **Reference Sequence Nucleotide** | **Reference Sequence Amino Acid** |
| --- | --- | --- | --- | --- | --- |
| RNA1 | 664 | **G** | ORF A: **A215** | **A** | ORF A: **T215** |
| RNA1 | 3129 | **A** | ORF B2: **Q129**  ORF A: **A1036** | **U** | ORF B2: **L129**  ORF A: **A1036** |
| RNA1 | 3179 | **U** | (3’UTR) | **A** | (3’UTR) |
| RNA2 | 328 | **G** | **T103** | **U** | **T103** |

**Table S2: Links to viral sequences used in this study:**

| Virus (protein) | Link |
| --- | --- |
| Nodamura virus (A/B2) | <http://ftp.cbi.pku.edu.cn/pub/database/Genome/Viruses/Nodamura_virus_uid14724/NC_002690.faa> |
| Nodamura virus (Capsid) | <http://ftp.cbi.pku.edu.cn/pub/database/Genome/Viruses/Nodamura_virus_uid14724/NC_002691.faa> |
| Striped Jack nervous necrosis virus (A/B) | <ftp://ftp.ncbi.nlm.nih.gov/genomes/Viruses/Striped_Jack_nervous_necrosis_virus_uid14741/NC_003448.faa> |
| Striped Jack nervous necrosis virus (Capsid) | <ftp://ftp.ncbi.nlm.nih.gov/genomes/Viruses/Striped_Jack_nervous_necrosis_virus_uid14741/NC_003449.faa> |
| Flock house virus (A/B1/B2) | <ftp://ftp.ncbi.nih.gov/genomes/Viruses/Flock_house_virus_uid15075/NC_004146.faa> |
| Flock house virus (Capsid) | <http://www.uniprot.org/uniprot/P12870> |
| Macrobrachium rosenbergii nodavirus (A/B2) | <http://ftp.cbi.pku.edu.cn/pub/database/Genome/Viruses/Macrobrachium_rosenbergii_nodavirus_uid15129/NC_005094.faa> |
| Macrobrachium rosenbergii nodavirus (Capsid) | <http://ftp.cbi.pku.edu.cn/pub/database/Genome/Viruses/Macrobrachium_rosenbergii_nodavirus_uid15129/NC_005095.faa> |
| Boolarra virus (A/B2) | <http://ftp.cbi.pku.edu.cn/pub/database/Genome/Viruses/Boolarra_virus_uid14850/NC_004142.faa> |
| Boolarra virus (Capsid) | <http://www.uniprot.org/uniprot/P12869> |
| Pariacoto virus (A/B2) | <http://www.metalife.com/Genbank/8050520> |
| Pariacoto virus (Capsid) | <http://www.uniprot.org/uniprot/Q9J7Z0> |
| Alphanodavirus HB-2007/CHN (A) | <http://www.uniprot.org/uniprot/D6PUS2> |
| Alphanodavirus HB-2007/CHN (Capsid) | <http://www.uniprot.org/uniprot/D6PUS1> |

**Table S3: Relative composition (% of each amino acid) in disordered regions:**

| residue | residue occurrence in predicted disordered regions in B1 from nodaviruses (in %) | Residue occurrence in predicted disordered regions of the entire uniprot (in %) |
| --- | --- | --- |
| **A** | 9.48 | 8.01 |
| **R** | 12.70 | 6.49 |
| **N** | 5.70 | 4.61 |
| **D** | 3.76 | 5.95 |
| **C** | 0.53 | 0.61 |
| **E** | 2.61 | 8.81 |
| **Q** | 5.72 | 5.28 |
| **G** | 13.64 | 7.32 |
| **H** | 1.93 | 2.49 |
| **I** | 1.38 | 3.66 |
| **L** | 2.38 | 6.61 |
| **K** | 6.29 | 6.47 |
| **M** | 1.22 | 2.00 |
| **F** | 0.10 | 1.92 |
| **P** | 11.03 | 7.29 |
| **S** | 10.70 | 8.54 |
| **T** | 8.17 | 6.43 |
| **W** | 0.10 | 0.62 |
| **Y** | 0.00 | 1.66 |
| **V** | 2.54 | 5.23 |

**Supporting Data References**

1. Albarino CG, Price BD, Eckerle LD, Ball LA (2001) Characterization and template properties of RNA dimers generated during flock house virus RNA replication. Virology 289: 269-282.

2. Johnson KL, Price BD, Ball LA (2003) Recovery of infectivity from cDNA clones of nodamura virus and identification of small nonstructural proteins. Virology 305: 436-451.

3. Dasgupta R, Sgro JY (1989) Nucleotide sequences of three Nodavirus RNA2's: the messengers for their coat protein precursors. Nucleic Acids Res 17: 7525-7526.

4. Byrne BJ, Davis MS, Yamaguchi J, Bergsma DJ, Subramanian KN (1983) Definition of the Simian Virus-40 Early Promoter Region and Demonstration of a Host Range Bias in the Enhancement Effect of the Simian Virus-40 72-Base-Pair Repeat. Proceedings of the National Academy of Sciences of the United States of America-Biological Sciences 80: 721-725.

5. Ball LA (1995) Requirements for the Self-Directed Replication of Flock House Virus-Rna-1. Journal of Virology 69: 720-727.

6. Wu HN, Lin YJ, Lin FP, Makino S, Chang MF, et al. (1989) Human Hepatitis-Delta Virus-Rna Subfragments Contain an Autocleavage Activity. Proceedings of the National Academy of Sciences of the United States of America 86: 1831-1835.
